# Prevalence of HPV, cytological abnormalities, and impact of the HPV vaccine in Mexico: a Nationwide Study of 596,944 women

**DOI:** 10.1016/j.lana.2025.101156

**Published:** 2025-06-25

**Authors:** Abraham García-Gil, Marco Antonio Luna-Ruiz-Esparza, José Luis Moreno-Camacho, Diana Yadira Calva-Espinosa, Ludwing Erick González-Mena, Luis Fernando Hernández-Lezama, Pablo Kuri-Morales, Juan Carlos Balcázar-Rodríguez, Abraham Campos-Romero, Jonathan Alcántar-Fernández

**Affiliations:** a Innovation and Research Department, Salud Digna, Culiacan, Sinaloa, 80000, Mexico; b Clinical Laboratory Department, Salud Digna, Culiacan, Sinaloa, 80000, Mexico; c National Reference Center II, Salud Digna, Tlalnepantla de Baz, Estado de Mexico, 54075, Mexico; d Faculty of Law, National Autonomous University of Mexico, Mexico City, 04510, Mexico; e Instituto Tecnológico y de Estudios Superiores de Monterrey, Monterrey, Nuevo Leon, 64700, Mexico; f Servicio de Colposcopia y Patología del Tracto Inferior del Hospital ISSSTE Dr. Manuel Cardenas de la Vega, Culiacan, Sinaloa, 8000, Mexico

**Keywords:** HPV, HPV vaccination, Cervical cancer, Cytological abnormalities

## Abstract

**Background:**

Cervical cancer (CC) remains a significant public health challenge worldwide and is the second leading cause of cancer-related death in women in Mexico. Although CC is highly preventable, effective implementation of screening programs to detect women with precancerous lesions is crucial to reduce its burden. This study evaluated HPV infection, its correlation with cytological abnormalities, and the impact of HPV vaccination in 596,944 women from all 32 states of Mexico.

**Methods:**

Samples were processed using a fully automated molecular biology laboratory setup (Roche Cobas prime - Cobas 6800), and cytotechnologists assessed cytological outcomes.

**Findings:**

The highest prevalence of HPV infection was observed among women under the age of 25, with 23,615 individuals (37.4%) testing positive. This was primarily attributable to infections with the pooled high-risk HPV genotypes included in the assay (HPV31, 33, 35, 39, 45, 51, 52, 56, 58, 59, 66, and 68), which accounted for 22,792 cases (36.1%). In contrast, HPV16 and HPV18 were most prevalent among women aged 25–34 years, with 7251 (5.21%) and 3281 (2.36%) infections, respectively. Low-grade squamous intraepithelial lesion (LSIL) was the most frequently detected cytological abnormality, identified in 15,411 cases (2.6%), and was predominantly associated with the pooled HPV genotypes (73.7%). A two-dose HPV vaccination regimen conferred strong protection against HPV16 (odds ratio [OR] = 0.21) and HPV18 (OR = 0.33), but did not significantly reduce the prevalence of infection with the pooled HPV genotypes (OR = 0.98).

**Interpretation:**

The notable carcinogenic potential of the HPV POOL underscores the need for broader vaccine formulations. Adopting a nonavalent vaccine in future campaigns, expanding screening coverage, and reinforcing sexual education for younger women are key measures to help policymakers mitigate the impact of CC in Mexico.

**Funding:**

This work was funded by Salud Digna.

**Research in context**

**Evidence before this study**

We conducted a literature search on PubMed, the World Health Organization, the Pan American Health Organization, the American Cancer Association, and The Global Cancer Observatory websites for information on cervical cancer and human papillomavirus (HPV). Additionally, we reviewed the Mexican Government’s official websites for national policies against cervical cancer and screened references to identify further relevant publications. Our search, which included information published up to October 2024, focused on terms such as HPV, HPV screening, HPV vaccination, cervical cytology, Papanicolaou, HPV prevalence, HPV Mexico, cervical cancer, cervical cancer Mexico, and cytological abnormalities.

**Added value of this study**

Our study is the first to provide a comprehensive national update on HPV infection dynamics across all 32 states of Mexico, involving a large cohort of women which enhances the robustness of our findings. We explored the distribution of high-risk HPV genotypes, their correlation with cytological abnormalities, and the impact of HPV vaccination programs. Notably, this is the first effort to evaluate the effect of HPV vaccination on circulating genotypes in Mexico. We confirmed a higher prevalence of HPV in younger women, predominantly non-HPV16 and non-HPV18 genotypes. Our findings also indicate that while HPV vaccines are effective against HPV16 and HPV18, they are less protective against other pooled genotypes, with enhanced protection observed in a two-dose vaccination scheme.

**Implications of all the available evidence**

The results underscore the need to strengthen population screening programs, particularly in states with limited screening access and among non-vaccinated women. There is also a pressing need to enhance vaccination strategies, ideally by adopting a nonavalent vaccine administered in a two-dose scheme. Further follow-up studies are essential to evaluate the long-term efficacy of HPV vaccination against cervical cancer, not just in Mexico but across other regions in the Americas with similar social and cultural dynamics. Policy efforts should prioritize areas with high HPV prevalence and low vaccination rates to mitigate the burden of cervical cancer effectively.

## Introduction

Cervical cancer (CC) ranks as the fourth most common cancer among women globally. In 2022, it was responsible for approximately 350,000 deaths, with around 660,000 new cases reported.^1^ In Mexico, CC is the second leading cause of cancer-related deaths among women. An estimated 49.6 million Mexican women are at risk of developing CC, with the disease contributing to 296 disability-adjusted life years (DALYs) per 100,000 persons annually as of 2019.^1^^,^^2^ CC originates from the abnormal and uncontrolled proliferation of epithelial cells in the cervix.^3^ The primary cause of CC is persistent infection with the human papillomavirus (HPV),^4^ which comprises over 200 genotypes. Of these, 12 are classified as high-risk by the World Health Organization due to their significant oncogenic potential, with HPV types 16 and 18 alone accounting for approximately 70% of CC cases worldwide.^5^

It is well established that most sexually active women will contract HPV at least once during their lifetime, yet the majority will not develop cervical cancer (CC).^6^ Most HPV infections resolve spontaneously within 12–24 months, but a small percentage persists and can lead to CC over a span of 10–15 years.^7^ The availability of HPV vaccination and the lengthy progression from persistent HPV infection to tumor development provide a critical window for the detection and treatment of high-grade lesions (CIN2+), making CC one of the most preventable forms of cancer.^7^ Consequently, the implementation of effective screening systems for early detection of CC is crucial. Knaul et al.^8^ emphasized that ‘cervical cancer is not a disease of the past—it is a disease of the poor,’ with 90% of CC cases occurring in low- and middle-income countries, primarily due to a lack of HPV vaccination, inadequate screening, and limited access to treatment. Despite these preventive measures, the global incidence of CC is expected to rise from 570,000 cases in 2018 to 700,000 by 2030, with deaths increasing from 311,000 to 400,000 during the same period.^9^

In November 2020, the WHO launched a Global strategy to eliminate cervical cancer (CC) as a public health problem by 2030, anchored by the 90-70-90 targets: 90% of girls fully vaccinated by age 15, 70% of women screened with a high-performance test by age 35 and again by 45, and 90% of women identified with cervical disease receiving treatment. Achieving these targets is projected to save 300,000 lives by 2030 and approximately 14 million lives by 2070.^9^ In response to this challenge, Salud Digna, a non-profit provider of diagnostic services in Mexico, initiated the ‘Aliados por la Salud’ campaign in partnership with the Bilbao Vizcaya Argentaria Bank (BBVA). Over six months, the campaign provided over 500,000 free cervical cancer screening tests, using real-time PCR for HPV detection and co-testing with liquid-based cytology tests. This effort not only increased access to early and accurate diagnoses but also generated significant epidemiological data that will enhance understanding of CC dynamics across the nation and support the development of targeted policies for its eradication. Through our study, we aim to analyze the prevalence of high-risk HPV, its correlation with cytological abnormalities, and the impact of HPV vaccination on the circulating genotypes across all 32 states of Mexico.

## Methods

For this study, the anonymized electronic health records of CC screening from all eligible patients responding to the “*Aliados por la Salud*” campaign who were attendants in the outpatient care clinics Salud Digna in all 32 states of Mexico from June 06, 2023, and 31 December 2023 were included. Available data collected during gynecological examinations, such as obstetric information and gynecologic and demographic data, including vaccination status, were included. A unique standardized and validated survey was applied for data collection in all clinics.

The exclusion criteria were the following: pregnant women and women with hysterectomy.

### Consent for using information, handling data, and protecting information privacy

The consent for using information from health records was obtained according to the Mexican Federal Law on Personal Data Protection (LFPDPPP, by its acronyms in Spanish). People who are attendants in the Salud Digna clinics accept our privacy policy, which includes the use of their information for scientific research purposes; by the above, we do not need specific informed consent from each person included in this work because this study is a cross-sectional analysis of an electronic health registry. We handled data protection and privacy according to national laws and guidelines in Mexico. Data obtained was anonymized by assigning a unique ID code to protect people’s identity and prevent data duplication in subsequent analysis.

### Ethical statement

This study was approved by the Ethical Review and Research Board of Salud Digna (SDI-20242). All methods adhered to the approved guidelines for clinical management information, the Declaration of Helsinki, and the country’s national regulations (Federal Law on Data Privacy Protection). Data were anonymized for study purposes.

### Procedures

CC screening results from the registry were obtained from the samples collected in the BD SurePath liquid-based cytology collectors during gynecological examinations in all clinics; the samples were then processed at the Cytology units and Molecular biology laboratories at the National Reference Centers of Salud Digna in the cities of Culiacan, Tlalnepantla, and Monterrey.

Cytology samples were processed in the BD Totalys MultiProcessor (BD Diagnostics, Sparks, USA) to enrich cervical cells (automated sample transfer, centrifugation, liquid decanting, and cell aspiration). The slide preparation and staining for the liquid-based cytology test were performed in the BD Totalys SlidePrep (BD Diagnostics, Sparks, USA) according to the manufacturer’s recommendations. Slides were visually inspected in Carl Zeiss Primo Star HD microscopes (Carl Zeiss Microscopy, LLC, USA) or by telecytology in samples digitized in the VENTANA DP 600 slide scanner (ROCHE Diagnostics, Basel, Switzerland) without the support of artificial intelligence by certified cytotechnologists; certified pathologists confirmed all abnormalities observed and 10% of all cytotechnologist-declared negative slides (pathologists were blinded for the cytotechnologist stated result). Liquid-based cytology test result was reported according to the 3rd ed. of The Bethesda System for Reporting Cervical Cytology.^10^

HPV DNA testing was performed using the fully automated molecular work area consisting of the analytical systems Cobas Prime and Cobas 6800, according to the manufacturer’s instructions. HPV genotyping was determined using the nucleic acid amplification test (NAAT) Cobas HPV test (ROCHE Diagnostics, Basel, Switzerland), which detects and amplifies HPV type-specific L1 gene and has been validated to be used for CC screening.^11^ This multiplex assay simultaneously detects 14 high-risk HPV types, HPV 16 and HPV 18 as independent results and 12 hr-HPV (HPV POOL:31, 33, 35, 39, 45, 51, 52, 56, 58, 59, 66, and 68).

A proportion of samples were processed in a semi-automated setup; briefly, nucleic acid was extracted in the automated extraction instrument Kingfisher Flex (Thermo Fisher Scientific, Waltham, Massachusetts) using the MagMAX Viral/Pathogen Nucleic Acid Isolation Kit (Thermo Fisher Scientific, Waltham, Massachusetts), HPV detection and genotyping was performed by RT-PCR using the kit Anyplex Ⅱ HPV HR Detection (Seegene Inc, Seoul, South Korea) which also detects and amplifies HPV type-specific L1 gene and has also been validated to be used for CC screening.^12^ RT-PCR was performed in a CFX96 thermocycler (BioRad, Hercules, USA).

### Statistical analysis

Descriptive statistics were performed on all data sets using Microsoft Office Excel. Additionally, we used logistic regression models to evaluate the association between predictor variables and a binary outcome (HPV infection) with adjustment for age in the logistic regression to compute the adjusted odds ratios (AOR). The analysis was implemented in R using the glm() function with a binomial family specification; stats package (v3.6.2) and R (v4.3.3) were used for logistic regression analysis.^13^ The threshold for statistical significance was established at a two-tailed *p*-value of <0.05. Confidence intervals (95% CI) were calculated using an exact binomial test for proportions. For reporting purposes, genotypes detected in the HPV POOL were aggregated and treated as a single unit.

### Role of the funding source

This work was funded by Salud Digna.

## Results

### Population characteristics

Between June 6, 2023, and December 31, 2023, a total of 596,944 women participated in cervical cancer (CC) screening at Salud Digna diagnostic clinics as part of the ‘Aliados por la Salud’ campaign. The average age of the participants was 42.2 ± 13.5 years. 483,460 (81%) of the women reported having been pregnant, 353,854 (59.3%) had their first sexual intercourse at age 18 or older, and 312,411 (52.3%) reported using a contraceptive method; of these, 160,982 (51.5%) had undergone bilateral tubal ligation surgery. 548,213 of the women (91.8%) reported not using tobacco, and 559,135 (93.7%) did not report having a sexually transmitted disease. Additionally, 277,012 (46.4%) participants indicated they did not have social security. A detailed summary of the demographic characteristics of the women included in this study is presented in Table 1.

**Table 1:** Demographic characteristics of women included in this study.

Characteristic	n	%
Age (years)		
Mean y ± SD	42.19 ± 13.5	
Range	14–97	
<25	63,147	10.6%
25–34	139,288	23.3%
35–44	127,303	21.3%
45–54	145,101	24.3%
55–64	92,685	15.5%
65–74	26,253	4.4%
≥75	3167	0.5%
Age at first sexual encounter (years)		
<18	222,407	37.3%
≥18	353,854	59.3%
Missed	20,683	3.5%
Use of contraceptive methods		
No	284,533	47.7%
Yes	312,411	52.3%
Condom	67,488	21.6%
Intrauterine device (IUD)	41,629	13.3%
Hormonal^a^	43,492	13.9%
Bilateral tubal ligation	160,982	51.5%
Fertility awarness (FAM)	2454	0.8%
History of pregnancies		
Yes	483,460	81%
No	113,484	19%
Previous pap test (≤5 years)		
Yes	320,363	53.7%
No	276,581	46.3%
HPV vaccination		
Yes	64,201	10.8%
No	532,743	89.2%
Tobacco consumption		
Yes	48,731	8.2%
No	548,213	91.8%
Sexually transmitted diseases		
Yes	37,809	6.3%
No	559,135	93.7%
Social security		
Yes	319,895	53.6%
IMSS	242,954	76%
INSABI	21,765	6.8%
ISSSTE	55,176	17.2%
No	277,012	46.4%
Private security services	29,506	10.6%
None	247,506	89.4%
Missed	37	0.01%

IMSS = acronyms in Spanish for The Mexican Institute of Social Security, INSABI = acronyms in Spanish for The Institute of Health for Well-being, ISSSTE = acronyms in Spanish for Institute for Social Security and Services for State Workers.

a Includes oral contraceptives, injections, implants, and patches.

### Prevalence of circulating HPV genotypes

The overall prevalence of HPV was 21.8%; the most prevalent genotypes were from the HPV POOL at 18.9%, followed by HPV16 at 3.4% and HPV18 at 1.7% (see Supplementary Table S1). The prevalence was higher in women who had their first sexual intercourse before the age of 18 years (24.9%) compared to those who had their first sexual intercourse at 18 years or older (20%) (Table 2). Similarly, women who reported consuming tobacco or having a sexually transmitted disease exhibited higher HPV prevalence rates, at 28.0% and 28.2%, respectively, compared to those who did not use tobacco (21.2%) or those without a sexually transmitted disease (21.4%) (see Table 2).

**Table 2:** Correlations between gynecologic, obstetrics and lifestyle characteristics with HPV infections.

Characteristic	n	HPV Positive (%)	Age adjusted OR (95% CI)	Unadjusted OR (95% CI)
**Social security**				
Yes	319,895	21.40%	0.95 (0.94–0.97)	0.94 (0.93–0.95)
No	277,012	22.40%	1	1
**Age at first sexual encounter (years)**				
<18	222,407	24.90%	1	1
≥18	353,854	20.00%	0.92 (0.91–0.93)	0.75 (0.74–0.76)
**Use of contraceptive methods**				
Yes	312,411	23.50%	1.06 (1.05–1.08)	1.24 (1.22–1.25)
No	284,533	19.90%	1	1
**Use of oral contraceptives**				
Yes	11,105	30.70%	1.08 (1.03–1.13)	1.61 (1.54–1.67)
No	585,839	21.60%	1	1
**History of pregnancies**				
Yes	483,460	19.30%	0.86 (0.84–0.89)	0.50 (0.49–0.50)
No	113,484	32.50%	1	1
**History of parity**				
Yes	289,995	18.60%	0.95 (0.93–0.97)	0.69 (0.68–0.70)
No	306,949	24.80%	1	1
**History of cesarian surgeries**				
Yes	253,729	18.20%	0.84 (0.83–0.86)	0.69 (068–0.69)
No	343,215	24.50%	1	1
**History of abortions**				
Yes	160,319	20.30%	1.05 (1.04–1.07)	0.88 (0.87–0.89)
No	436,625	22.40%	1	1
**Tobacco consumption**				
Yes	48,731	28.00%	1.26 (1.23–1.28)	1.45 (1.42–1.48)
No	548,213	21.20%	1	1
**Sexually transmitted diseases**				
Yes	37,809	28.20%	1.37 (1.33–1.41)	1.44 (1.41–1.48)
No	559,135	21.40%	1	1
**Current infection with *Candida***				
Yes	4132	39.60%	1.43 (1.34–1.53)	2.37 (2.22 2.52)
No	592,812	21.70%	1	1
**Current infection with *Trichomona vaginalis***				
Yes	246	28.90%	0.92 (0.69–1.22)	1.46 (1.11–1.92)
No	596,698	21.80%	1	1
**Current infection with *Gardnerella vaginalis***				
Yes	698	29.90%	1.01 (0.86–1.20)	1.53 (1.30–1.80)
No	596,246	21.80%	1	1
**Current infection with *Herpes simple***				
Yes	686	32.20%	1.08 (0.91–1.27)	1.70 (1.45–2.00)
No	596,258	21.80%	1	

Odds ratio (OR) were adjusted by age (included in the model as a categorical variable; <25, 25–34, 35–44, 45–54, 55–64, 65–74, ≥75).

CI = Confidence interval; HPV = Human papillomavirus.

### Prevalence of hr-HPV genotypes in México

**Fig. 1:**
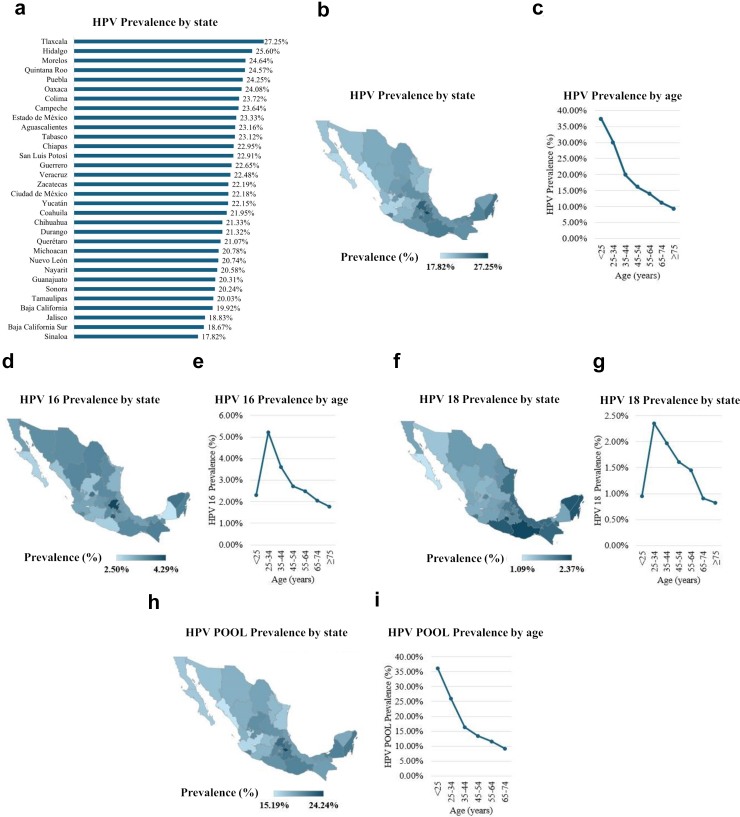
HPV prevalence in Mexico by state **(a)**. Choropleth map of HPV prevalence in Mexico **(b)**. HPV prevalence in Mexico by age **(c)**. Choropleth map of HPV 16 prevalence in Mexico **(d)**. HPV 16 prevalence in Mexico by age **(e)**. Choropleth map of HPV 18 prevalence in Mexico **(f)**. HPV 18 prevalence in Mexico by age **(g)**. Choropleth map of HPV POOL prevalence in Mexico **(h)**. HPV POOL prevalence in Mexico by age **(i)**.

The highest hr-HPV prevalence was observed in Tlaxcala (27.3%) and Hidalgo (25.6%), while the lowest was in Sinaloa (17.8%) and Baja California Sur (18.7%) (Fig. 1a and b). For HPV 16, Hidalgo (4.3%) and Morelos (4%) had the highest rates, with the lowest in Campeche (2.5%) and Baja California Sur (2.7%) (Fig. 1d, Supplementary Table S1). The highest prevalence of HPV 18 was found in Oaxaca (2.4%) and Morelos (2.3%), whereas the lowest was in Baja California Sur (1.1%) and Sonora (1.4%) (Fig. 1f, Supplementary Table S1). Tlaxcala (24.3%) and Hidalgo (22.1%) had the highest HPV POOL prevalence, in contrast to Sinaloa (15.2%) and Jalisco (15.9%) which had the lowest (Fig. 1h, Supplementary Table S1).

### Age distribution of HPV

The highest HPV prevalence was observed in women younger than 25 years (37.4%), with prevalence decreasing with age (Fig. 1c). Women aged 25–34 years exhibited the highest prevalence of HPV 16 and HPV 18 (5.2% and 2.3% respectively) (Fig. 1e and g, Supplementary Table S2), followed by those aged 35–44 years (3.6% and 2% respectively) (Fig. 1e and g, Supplementary Table S2). Similarly, the highest HPV POOL prevalence was found in women younger than 25 years (36.1%), with a decreasing trend as the population ages (Fig. 1i, Supplementary Table S2).

### Cytological abnormalities prevalence and hr-HPV genotypes correlation

**Fig. 2:**
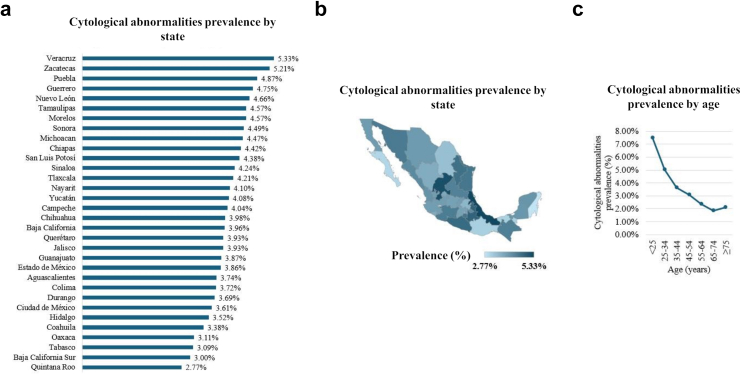
Cytological abnormalities prevalence in Mexico by state **(a)**. Choropleth map of cytological abnormalities prevalence in Mexico **(b)**. Cytological abnormalities prevalence in Mexico by age **(c)**.

The overall prevalence of cytological abnormalities was 3.9%, with LSIL being the most prevalent lesion at 2.6%, followed by ASCUS at 0.9% (Supplementary Table S3). The highest prevalence of cytological abnormalities was observed in Veracruz (5.3%) and Zacatecas (5.2%), while the lowest was in Quintana Roo (2.8%) and Baja California Sur (3%) (Fig. 2a, Supplementary Table S3). The prevalence of cytological abnormalities showed a similar age-related trend as HPV, with the highest prevalence found in women under 25 years old (37.4%), decreasing with age (Fig. 2c, Supplementary Table S4).

**Fig. 3:**
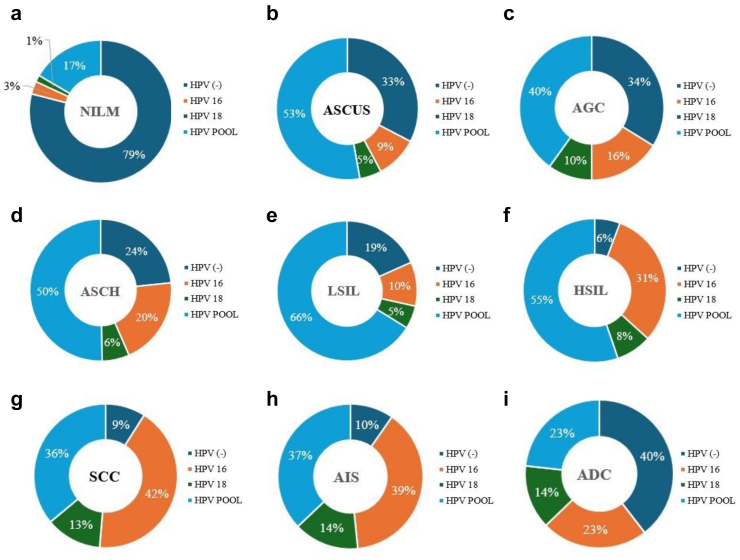
Cytological abnormalities and their correlation to HPV genotypes infection. Negative for intraepithelial lesions or malignancy (NILM) **(a)**. Atypical squamous cells with undetermined significance (ASCUS) **(b)**. Atypical glandular cells (AGC) **(c)**. Atypical squamous cells cannot exclude high-grade squamous intraepithelial lesions (ASCH) **(d)**. Low-grade squamous intraepithelial lesions (LSIL) **(e)**. High-grade squamous intraepithelial lesions (HSIL) **(f)**. Squamous cell carcinoma (SCC) **(g)**. Adenocarcinoma *in situ* (AIS) **(h)** Adenocarcinoma (ADC) **(i)**.

Regarding the correlation of HPV genotypes with cytological abnormalities, the prevalence of HPV in women with negative intraepithelial lesions or malignancy (NILM) was 19.6%. This prevalence increased significantly in more severe cytological outcomes: 64.7% in ASCUS, 61.9% in atypical glandular cells (AGC), 73.6% in atypical squamous cells that cannot exclude HSIL (ASC-H), 79.6% in LSIL, 93.1% in HSIL, 89.8% in squamous cell carcinoma (SCC), 88% in adenocarcinoma *in situ* (AIS), and 56.4% in adenocarcinoma (ADC) (Supplementary Table S5). The genotypes within the HPV POOL were most frequently detected in NILM (16.8%), ASCUS (57.2%), AGC (45.2%), ASC-H (57%), LSIL (73.7%), and HSIL (66.3%). In more advanced lesions, a mixed pattern was observed between the HPV POOL and HPV 16: SCC (HPV POOL 40.8%, HPV 16 48%), AIS (HPV POOL 46%, HPV 16 48%), and ADC (HPV POOL 25.6%, HPV 16 25.6%). Throughout all cytological abnormalities, HPV 18 was the least prevalent genotype (Fig. 3a–i).

### Risk factors association to HPV infection

Based on data collected through surveys completed during HPV-RT PCR and liquid-based cytology co-testing, we analyzed the correlation between various obstetric and gynecological characteristics and HPV infection. A significant association was found with tobacco consumption (OR = 1.26; CI = 1.23–1.28), sexually transmitted diseases (OR = 1.37; CI = 1.33–1.41), and Candida infections (OR = 1.43; CI = 1.34–1.53), which increased the odds of contracting HPV (Table 2).

### HPV vaccination and HPV

**Fig. 4:**
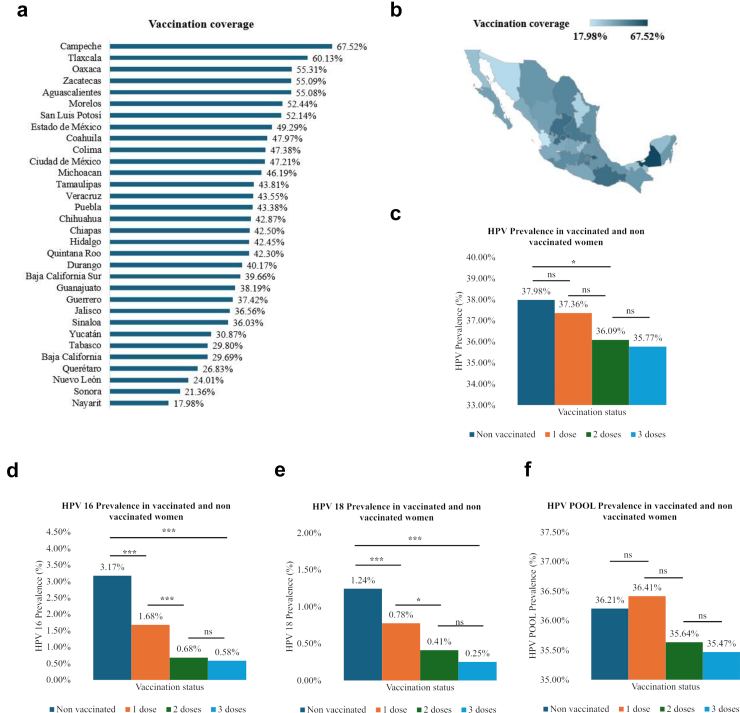
HPV Vaccination coverage by state **(a)**. Choropleth map of HPV vaccination coverage **(b)**. HPV prevalence in vaccinated and non-vaccinated women **(c)**. HPV 16 prevalence in vaccinated and non-vaccinated women **(d)**. HPV 18 prevalence in vaccinated and non-vaccinated women **(e)**. HPV POOL prevalence in vaccinated and non-vaccinated women **(f)**. Significative differences: ∗*p* < 0.05, ∗∗*p* < 0.01, ∗∗∗*p* < 0.001.

As emphasized by Kombe et al.^14^ “Epidemiological surveillance of HPV infection and related diseases represents a crucial topic for monitoring and evaluation of the three currently available antiviral prophylactic vaccines”. Following the WHO recommendations for HPV vaccination, and in line with Mexico’s national vaccination program initiated in 2012, we focused on the vaccination’s impact among women under 25 years of age (n = 63,147). The vaccination coverage in this group was 41.6%, with 17.4% having received one dose (1D), 18.6% two doses (2D), and 5.7% three doses (3D) (Supplementary Table S6). The highest vaccination rates were in Campeche (67.5%) and Tlaxcala (60.1%), while the lowest were in Nayarit (18%) and Sonora (21.3%) (Fig. 4a and b). Notably, the prevalence of HPV 16 was lower in vaccinated women compared to non-vaccinated women (NV 3.2%, 1D 1.7%, 2D 0.7%, 3D 0.6%). A similar trend was observed for HPV 18 (NV 1.2%, 1D 0.8%, 2D 0.4%, 3D 0.3%), though no significant trend was noted in the HPV POOL prevalence (NV 36.2%, 1D 36.4%, 2D 35.6%, 3D 35.5%) (Fig. 4d–f). Our odds ratio analysis indicates a protective effect of vaccination against HPV 16 (1D OR = 0.52, CI = 0.44–0.61; 2D OR = 0.21, CI = 0.17–0.26; 3D OR = 0.18, CI = 0.11–0.26) and HPV 18 (1D OR = 0.62, CI = 0.49–0.78; 2D OR = 0.33, CI = 0.24–0.44; 3D OR = 0.20, CI = 0.10–0.36), but not against HPV POOL infection (1D OR = 1.01, CI = 0.97–1.05; 2D OR = 0.98, CI = 0.93–1.02; 3D OR = 0.97, CI = 0.90–1.04) (Supplementary Table S7).

## Discussion

The ‘Aliados por la Salud’ campaign has had a significant impact on Mexico, marking a unique initiative in Latin America. Given the limited access to HPV molecular screening tests in the public sector, this campaign has spurred vital discussions about cervical cancer and encouraged health professionals to familiarize themselves with new diagnostic methods, particularly nucleic acid amplification tests (NAAT) such as RT-PCR.

Our study leveraged the extensive data provided by the campaign, covering a substantial number of patients across all 32 states of Mexico. This data has been instrumental in understanding the dynamics of high-risk HPV (hr-HPV) and its correlation with cytological abnormalities that could progress to cervical cancer (CC). The study population, consisting of women undergoing screening at Salud Digna clinics as part of the campaign, notably includes 46.4% who lack access to social security services, highlighting their increased vulnerability to CC.

We report a national HPV prevalence of 21.8%. This is a slight decrease from the 24.8% prevalence our group reported in 2019,^15^ and significantly higher than the 10.3% reported by the Mexican National Center for Gender Equity and Health in the same year.^16^ These discrepancies can be attributed to differences in the technologies used for sample processing, the range of genotypes detected, and the inclusion of age groups typically not screened in the public sector, which focuses on women over 35. Notably, data from 2019 indicated an HPV 16 prevalence of over 8% and an HPV 18 prevalence of over 3%,^15^ whereas our current findings show an HPV 16 prevalence of 2.3% and an HPV 18 prevalence of 0.95%. This reduction may reflect the impact of vaccination among younger women; however, more detailed studies are necessary to fully evaluate the effects of immunization on HPV prevalence among Mexican women.

Consistent with global reports,^14^^,^^17^ our data indicates that women in Mexico are infected with HPV at an early age (<25 years), likely due to sexual behaviors and anatomical features—during adolescence, the cervix’s transformation zone is more active, allowing easier access for HPV to epithelial cells. We observed a higher prevalence of HPV in young women, which decreases as they age due to the natural clearance of the infection. Remarkably, HPV 16 and HPV 18, the most carcinogenic genotypes, increase in prevalence among women in their reproductive and economically active years (25–44 years old). This underscores the need to enhance screening programs to identify women with persistent HPV infections. Similarly, while the prevalence of cytological abnormalities decreases with age, LSIL remains the most common in younger women. However, more severe cytological abnormalities such as HSIL, SCC, AIS, and ADC tend to increase with age in women with persistent HPV infections, correlating with the higher prevalence of HPV 16 and HPV 18 in these cases.^14^

Furthermore, despite a general decrease in the prevalence of the genotypes within the HPV POOL as the population ages, these genotypes still significantly contribute to cytological abnormalities, demonstrating considerable carcinogenic potential. Intriguingly, our analysis revealed that 9.0% of SCC, 9.7% of AIS, and 39.5% of ADC cases were HPV-negative. This could be partially explained by the loss of the L1/E1 regions—the targets of RT-PCR primers—during the integration of viral DNA into the host genome.^18^ Additionally, it has been reported that between 2001 and 2008, up to 7.1% of women with cervical cancer had a negative HPV result.^19^ The phenomenon of HPV-negative cervical cancer remains poorly understood, with its etiology still elusive, and the possibility of false-negative HPV results cannot be dismissed.

It is important to mention a technical limitation associated with the HPV POOL; we cannot individually associate any HPV genotypes clustered in the HPV POOL with a cytological abnormality. It has been reported that the HPV genotypes most frequently found in cervical cancers in Mexico besides HPV 16 and HPV 18, were HPV 45, HPV 52, HPV 58, and HPV 39.^20^ Furthermore, previous analyses from our group have shown that the members of the alpha 9 genus (e.g., HPV 16/31 and HPV 33/58), are more prevalent in cytological abnormalities including cervical cancer.^15^ Further studies must be performed to assess the impact of the individual HPV genotypes pooled in our research on the Mexican population.

Mexico established a pilot HPV vaccination program in 2012 for girls in the fifth grade of primary school and 11-year-old girls not enrolled in school; in 2014, the program was officially established with a two-immunization scheme (0 and 6 months). In 2022, the vaccination program was modified to a one-immunization scheme to increase accessibility and vaccination coverage.^21^^,^^22^ Considering the beginning of the HPV vaccination program in Mexico 12 years ago, its impact on the circulating HPV genotypes should already be observable in vaccinated women. As expected, we observed a substantial reduction in the prevalences of the highest-risk genotypes (HPV 16 and HPV 18) but not in the prevalence of HPV POOL, whose genotypes are not targeted by the vaccines used in the vaccination programs.

We realize many countries are moving to a single-dose immunization scheme based on data reporting comparable protection between one or two dose schemes. However, our research’s statistical analysis showed us that there is a statistical difference between vaccinating with two doses instead of only one, hence supporting a two-dose vaccination scheme as recommended by the WHO.^23^ Further analysis must be performed on the Mexican population to evaluate HPV vaccination’s impact on circulating HPV genotypes.

Regarding vaccination coverage, our results must be taken cautiously because data has a self-declared origin. Nevertheless, there is no official national register of HPV-vaccinated women by state. Mongua-Rodriguez et al.^24^ based on the 2021 National Health and Nutrition Survey results, a 43.7% HPV vaccination rate in México was reported, similar to the rate we reported in this study (41.6%), with the same memory bias as ours. Moreover, in 2023, the WHO officially reported that 57.8% of women in Mexico received the last HPV vaccination dose.^25^

It is important to mention that we assessed the vaccine’s protection against HPV infection, not against the development of cervical lesions, we did not observe a correlation between vaccination status and cytological abnormalities, probably because our study on vaccination impact focused on young women, where critical cytological abnormalities have a low prevalence. In this case, it is important to implement follow-up studies to assess vaccination’s impact on the prevalence of invasive lesions.

Vaccination is the most important preventive measure against HPV infection, notably, it has been documented that female vaccination has an impact on HPV-related cancers and lesions in unvaccinated women and males through herd immunity,^23^^,^^26^ which could have significant implications in CC and non-cervical HPV-related cancers such as anal cancer and head and neck cancer. Studies assessing the impact of female HPV vaccination in males and non-vaccinated women in Mexico are required to deepen our understanding of the true impact vaccination has on HPV-related lesions and cancer beyond CC.

It is important to strengthen the vaccination programs, without missing that current vaccines do not protect against all HPV genotypes,^26^ hence, it is important to reinforce the screening programs mainly for women older than the vaccine-eligible group. Moreover, CC screening costs could be markedly reduced by ensuring high HPV vaccination coverage, resulting in a drop in critical cytological abnormalities incidence and colposcopy referrals.^27^

Even though the nonavalent vaccine is available in Mexico, it is not officially used in HPV vaccination campaigns; however, our results strongly suggest the implications of the genotypes belonging to the HPV POOL in cervical lesions; Serrano et al.^28^ Reported that the additional HPV 31/33/45/52/58 genotypes included in the nonavalent vaccine could increase 11.3% the protection against CC in México. Thus, we consider it important that policymakers consider this vaccine to be indicated for future anti-HPV vaccine campaigns.

This study provides valuable insights into the current national landscape of HPV infections and their association with cytological abnormalities. However, certain limitations must be acknowledged. The study population comprises individuals who voluntarily sought diagnostic services at Salud Digna, representing a self-selected group that includes both insured and uninsured individuals across diverse socioeconomic backgrounds. While this self-selection introduces potential bias, it is partially mitigated by the large volume of data collected, which enables more granular analyses under specific conditions. Consequently, findings derived from Salud Digna data should be interpreted within the context of this particular population and with consideration of the inherent selection bias.

### Conclusion

Our study underscores the urgent need to intensify efforts to eliminate cervical cancer (CC) as a public health challenge in Mexico. Despite global initiatives suggesting the feasibility of eradicating CC, our findings highlight the critical gaps in screening and vaccination coverage within a culturally diverse landscape. It is crucial to enhance screening programs, boost individual responsibility, increase self-care awareness, and significantly reduce the stigma associated with CC. To achieve these goals, robust multi-sectorial partnerships are essential, bridging the gap between private and public sectors to develop strategies that improve access to health services and early cancer diagnosis for women.

Self-sampling methodologies, which allow women to collect vaginal samples themselves, have been shown to offer diagnostic accuracy comparable to clinician-collected samples. This approach could dramatically increase screening coverage, particularly for women who lack access to traditional healthcare settings, by offering convenience, privacy, and emotional comfort.^29^, ^30^, ^31^, ^32^

Moreover, our data indicate that current vaccination rates are well below the WHO’s target of 90% coverage. As vaccination is the most effective tool to prevent CC, it is imperative to expand vaccination campaigns, incorporating multivalent vaccines to achieve broader geographical and demographic reach, particularly in rural areas and among populations at high risk.

Our study emphasizes:

The critical importance of utilizing vaccines that cover genotypes significantly involved in the development of carcinogenic cytological abnormalities. This supports the need for vaccines with broader genotype coverage to effectively reduce the incidence of cervical cancer.

The significant impact of vaccination strategies on public health outcomes. Our findings underscore the efficacy of the 2014 policy that introduced a two-immunization vaccination scheme, suggesting that reducing the number of doses may compromise protective benefits.

The effectiveness of targeted campaigns and innovative screening methods, such as self-sampling, in areas with high HPV prevalence. These strategies not only help in addressing the spread of the virus but also promote self-care practices among the young female population in Mexico.

The need for policymakers to focus on improving health strategies that place the well-being of the population at the forefront, ensuring that health policies effectively cater to the needs of vulnerable communities.

## Contributors

The current study was conceptualized and designed by G-GA, C-RA, and A-FJ. G-GA and L-R-EMA conducted data curation. G-GA and A-FJ conducted formal analysis. G-GA wrote the original manuscript draft, and addressed editions suggested by the rest of the authors. C-RA and A-FJ oversaw all analyses, offering comprehensive insights to refine principal and sensitivity analyses. G-GA and A-FJ directly accessed and verified the underlying data reported in the manuscript. G-GA and A-FJ coordinated the submission and peer review processes. L-R-EMA, M-CJL, C-EDY, G-MLE, H-LLF, K-MP, C-RA, A-FJ, and B-RJC critically reviewed the manuscript, tables, and figures for pertinent subject matter knowledge content and approved the final version.

## Data sharing statement

All relevant data is available in the manuscript and Supplementary information. Data is available upon reasonable request.

## Editor note

The Lancet Group takes a neutral position with respect to territorial claims in published maps and institutional affiliations.

## Declaration of interests

The authors declare no conflict of interest.
