# Supplementary material for: Prevalence of HPV, cytological abnormalities, and impact of the HPV vaccine in Mexico: a Nationwide Study of 596,944 women
Source: Lancet Reg Health Am. 2025 Jun 25;48:101156. doi: 10.1016/j.lana.2025.101156 (PMC12246922; doi:10.1016/j.lana.2025.101156)
Supplement: Supplementary Tables S1–S7 [file mmc1.pdf]

PREVALENCE OF HPV, CYTOLOGICAL ABNORMALITIES, AND IMPACT OF THE HPV VACCINE IN MEXICO: A NATIONWIDE STUDY OF 596,944 WOMEN

Abraham García-Gil PhD[1], Marco Antonio Luna-Ruiz-Esparza MPH[1], José Luis Moreno-Camacho MSc[2], Diana Yadira Calva-Espinosa MD[2], Ludwing Erick González-Mena MD[3], Luis Fernando Hernández-Lezama MBA[4], Pablo Kuri-Morales MD[5], Juan Carlos Balcázar-Rodríguez MD[6], Abraham Campos-Romero MD[1], Jonathan Alcántar-Fernández PhD[1]

[1]Innovation and Research Department, Salud Digna, Culiacan, 80000, Sinaloa, Mexico.

[2]Clinical Laboratory Department, Salud Digna, Culiacan, 80000, Sinaloa, Mexico.

[3]National Reference Center II, Salud Digna, Tlalnepantla de Baz, 54075, Estado de Mexico, Mexico.

[4]Faculty of Law, National Autonomous University of Mexico, Mexico City, 04510, Mexico.

[5]Instituto Tecnológico y de Estudios Superiores de Monterrey, Monterrey, 64700, Nuevo Leon, Mexico.

[6]Servicio de Colposcopia y Patología del tracto inferior del Hospital ISSSTE Dr. Manuel Cardenas de la Vega, Culiacan, 8000, Sinaloa, Mexico.

Supplementary material

State	n	Prevalence of HPV infections % (95% CI)			
		Overall	HPV 16	HPV 18	HPV POOL
Aguascalientes	5,251	23.16% (22.02% - 24.32%)	3.81% (3.31% - 4.36%)	1.85% (1.50% - 2.25%)	19.94% (18.87% - 21.05%)
Baja California	26,602	19.92% (19.44% - 20.40%)	3.29% (3.08% - 3.51%)	1.62% (1.48% - 1.78%)	16.93% (16.49% - 17.39%)
Baja California Sur	2,105	18.67% (17.03% - 20.40%)	2.71% (2.06% - 3.49%)	1.09% (0.69% - 1.64%)	16.82% (15.24% - 18.48%)
Campeche	1,362	23.64% (21.41% - 25.99%)	2.50% (1.73% - 3.47%)	1.62% (1.01% - 2.44%)	20.93% (18.79% - 23.18%)
Chiapas	11,440	22.95% (22.18% - 23.73%)	3.65% (3.31% - 4.00%)	2.11% (1.85% - 2.39%)	19.30% (18.58% - 20.04%)
Chihuahua	15,969	21.33% (20.70% - 21.97%)	3.50% (3.22% - 3.80%)	1.70% (1.50% - 1.91%)	18.22% (17.63% - 18.83%)
Ciudad de México	94,294	22.18% (21.92% - 22.45%)	3.27% (3.15% - 3.38%)	1.64% (1.56% - 1.72%)	19.36% (19.11% - 19.62%)
Coahuila	9,262	21.95% (21.11% - 22.81%)	3.58% (3.22% - 3.98%)	1.68% (1.43% - 1.97%)	18.96% (18.17% - 19.77%)
Colima	2,964	23.72% (22.20% - 25.29%)	3.54% (2.91% - 4.27%)	1.96% (1.49% - 2.52%)	20.55% (19.10% - 22.05%)
Durango	9,364	21.32% (20.49% - 22.16%)	3.57% (3.20% - 3.96%)	1.56% (1.32% - 1.83%)	18.29% (17.52% - 19.09%)
Estado de México	118,348	23.33% (23.09% - 23.57%)	3.51% (3.41% - 3.62%)	1.77% (1.69% - 1.84%)	20.47% (20.24% - 20.70%)
Guanajuato	27,730	20.31% (19.84% - 20.79%)	3.38% (3.17% - 3.60%)	1.63% (1.48% - 1.79%)	17.59% (17.14% - 18.04%)
Guerrero	4,433	22.65% (21.42% - 23.91%)	2.75% (2.29% - 3.28%)	2.21% (1.80% - 2.69%)	19.96% (18.80% - 21.17%)
Hidalgo	4,364	25.60% (24.31% - 26.92%)	4.29% (3.70% - 4.93%)	1.97% (1.58% - 2.43%)	22.11% (20.89% - 23.37%)
Jalisco	33,441	18.83% (18.41% - 19.25%)	3.23% (3.04% - 3.42%)	1.54% (1.41% - 1.68%)	15.93% (15.54% - 16.32%)
Michoacán	12,762	20.78% (20.08% - 21.49%)	3.02% (2.73% - 3.34%)	1.63% (1.42% - 1.86%)	18.05% (17.39% - 18.73%)
Morelos	4,022	24.64% (23.31% - 26.00%)	4.03% (3.44% - 4.68%)	2.29% (1.85% - 2.80%)	21.13% (19.88% - 22.43%)
Nayarit	5,059	20.58% (19.47% - 21.72%)	3.30% (2.83% - 3.83%)	1.60% (1.27% - 1.99%)	18.15% (17.09% - 19.24%)
Nuevo León	31,376	20.74% (20.29% - 21.19%)	3.51% (3.31% - 3.72%)	1.76% (1.62% - 1.91%)	17.71% (17.29% - 18.14%)
Oaxaca	3,845	24.08% (22.74% - 25.47%)	3.54% (2.98% - 4.17%)	2.37% (1.91% - 2.90%)	20.86% (19.58% - 22.18%)
Puebla	33,474	24.25% (23.79% - 24.71%)	3.35% (3.16% - 3.54%)	1.85% (1.71% - 2.00%)	21.47% (21.03% - 21.92%)
Querétaro	16,507	21.07% (20.45% - 21.70%)	3.51% (3.24% - 3.81%)	1.74% (1.55% - 1.96%)	18.02% (17.43% - 18.61%)
Quintana Roo	8,185	24.57% (23.64% - 25.52%)	3.64% (3.25% - 4.07%)	2.25% (1.94% - 2.59%)	21.33% (20.45% - 22.24%)
San Luis Potosí	7,723	22.91% (21.97% - 23.86%)	3.52% (3.12% - 3.96%)	1.55% (1.29% - 1.86%)	19.89% (19.00% - 20.80%)
Sinaloa	28,451	17.82% (17.37% - 18.27%)	2.90% (2.70% - 3.10%)	1.36% (1.23% - 1.51%)	15.19% (14.77% - 15.61%)
Sonora	19,103	20.24% (19.68% - 20.82%)	3.48% (3.22% - 3.75%)	1.36% (1.20% - 1.54%)	17.33% (16.79% - 17.87%)
Tabasco	7,724	23.12% (22.19% - 24.08%)	3.46% (3.06% - 3.89%)	2.08% (1.78% - 2.43%)	19.65% (18.77% - 20.56%)
Tamaulipas	14,112	20.03% (19.37% - 20.70%)	3.06% (2.78% - 3.36%)	2.03% (1.81% - 2.28%)	16.89% (16.28% - 17.52%)
Tlaxcala	2,484	27.25% (25.51% - 29.05%)	3.74% (3.03% - 4.57%)	2.05% (1.53% - 2.69%)	24.24% (22.56% - 25.97%)
Veracruz	24,430	22.48% (21.96% - 23.01%)	3.29% (3.07% - 3.52%)	2.08% (1.90% - 2.27%)	19.26% (18.77% - 19.76%)
Yucatán	7,770	22.15% (21.23% - 23.09%)	3.77% (3.36% - 4.22%)	2.24% (1.92% - 2.59%)	18.69% (17.83% - 19.57%)
Zacatecas	2,988	22.19% (20.71% - 23.72%)	2.88% (2.31% - 3.54%)	1.47% (1.07% - 1.97%)	20.08% (18.66% - 21.56%)
**Total**	596,944	21.80% (21.70% - 21.91%)	3.38% (3.33% - 3.42%)	1.73% (1.70% - 1.77%)	18.88% (18.78% - 18.98%)

Supplementary table 1. Overall HPV, HPV 16, HPV 18, and HPV POOL genotypes prevalence in Mexico and its states. Abreviatures: CI = Confidence interval. HPV = Human papillomavirus.

Age (years)	n	Prevalence of HPV infections % (95% CI)			
		Overall	HPV 16	HPV 18	HPV POOL
<25	63,147	37.41% (37.02% - 37.78%)	2.30% (2.19% - 2.42%)	0.95% (0.88% - 1.03%)	36.10% (35.72% - 36.47%)
25-34	139,288	29.98% (29.74% - 30.22%)	5.21% (5.09% - 5.32%)	2.36% (2.28% - 2.44%)	26.10% (25.86% - 26.32%)
35-44	127,303	19.86% (19.63% - 20.07%)	3.62% (3.52% - 3.72%)	1.97% (1.89% - 2.05%)	16.32% (16.11% - 16.52%)
45-54	145,101	16.08% (15.89% - 16.27%)	2.72% (2.63% - 2.80%)	1.61% (1.55% - 1.68%)	13.40% (13.23% - 13.58%)
55-64	92,685	14.00% (13.78% - 14.23%)	2.50% (2.40% - 2.60%)	1.45% (1.37% - 1.53%)	11.53% (11.32% - 11.73%)
65-74	26,253	11.12% (10.73% - 11.50%)	2.07% (1.90% - 2.24%)	0.91% (0.80% - 1.03%)	9.25% (8.90% - 9.61%)
≥75	3,167	9.28% (8.29% - 10.35%)	1.77% (1.34% - 2.29%)	0.82% (0.54% - 1.20%)	7.39% (6.50% - 8.36%)
**Total**	596,944				

Supplementary table 2. Overall HPV, HPV 16, HPV 18, and HPV POOL genotypes prevalence in Mexico by age. Abreviatures: CI = Confidence interval. HPV = Human papillomavirus.

State	n	Prevalence of cytological abnormalities % (95% CI)								
		NILM	ASCUS	AGC	ASCH	LSIL	HSIL	SCC	AIS	ADC
Aguascalientes	5,251	95.62% (95.03% - 96.16%)	1.31% (1.02% - 1.66%)	0.17% (0.08% - 0.33%)	0.08% (0.02% - 0.19%)	2.30% (1.92% - 2.75%)	0.46% (0.29% - 0.68%)	0.02% (0.00% - 0.11%)	0.02% (0.00% - 0.11%)	0.02% (0.00% - 0.11%)
Baja California	26,602	95.43% (95.17% - 95.68%)	1.36% (1.22% - 1.50%)	0.08% (0.05% - 0.13%)	0.15% (0.11% - 0.21%)	2.48% (2.30% - 2.68%)	0.43% (0.35% - 0.51%)	0.03% (0.01% - 0.06%)	0.02% (0.01% - 0.04%)	0.02% (0.00% - 0.04%)
Baja California Sur	2,105	95.34% (94.36% - 96.20%)	1.38% (0.92% - 1.97%)	0.05% (0.00% - 0.26%)	0.29% (0.10% - 0.62%)	2.61% (1.97% - 3.39%)	0.29% (0.10% - 0.62%)	0.00% (0.00% - 0.18%)	0.05% (0.00% - 0.26%)	0.00% (0.00% - 0.18%)
Campeche	1,362	94.79% (93.47% - 95.91%)	0.88% (0.46% - 1.53%)	0.00% (0.00% - 0.27%)	0.07% (0.00% - 0.41%)	3.67% (2.74% - 4.81%)	0.59% (0.25% - 1.15%)	0.00% (0.00% - 0.27%)	0.00% (0.00% - 0.27%)	0.00% (0.00% - 0.27%)
Chiapas	11,440	96.02% (95.65% - 96.37%)	0.68% (0.54% - 0.85%)	0.06% (0.02% - 0.13%)	0.06% (0.02% - 0.13%)	2.88% (2.58% - 3.20%)	0.29% (0.20% - 0.40%)	0.01% (0.00% - 0.05%)	0.00% (0.00% - 0.03%)	0.00% (0.00% - 0.03%)
Chihuahua	15,969	96.62% (96.33% - 96.90%)	0.85% (0.71% - 1.00%)	0.08% (0.04% - 0.14%)	0.16% (0.11% - 0.24%)	1.92% (1.72% - 2.15%)	0.31% (0.23% - 0.41%)	0.03% (0.01% - 0.06%)	0.03% (0.01% - 0.06%)	0.00% (0.00% - 0.02%)
Ciudad de México	94,294	96.28% (96.15% - 96.40%)	0.56% (0.51% - 0.61%)	0.05% (0.04% - 0.07%)	0.08% (0.06% - 0.09%)	2.80% (2.70% - 2.91%)	0.23% (0.20% - 0.26%)	0.01% (0.00% - 0.01%)	0.00% (0.00% - 0.01%)	0.00% (0.00% - 0.01%)
Coahuila	9,262	96.48% (96.08% - 96.85%)	0.69% (0.53% - 0.88%)	0.10% (0.04% - 0.18%)	0.06% (0.02% - 0.14%)	2.32% (2.02% - 2.65%)	0.31% (0.21% - 0.45%)	0.01% (0.00% - 0.06%)	0.00% (0.00% - 0.04%)	0.02% (0.00% - 0.08%)
Colima	2,964	96.39% (95.65% - 97.03%)	0.74% (0.47% - 1.12%)	0.07% (0.01% - 0.24%)	0.00% (0.00% - 0.12%)	2.56% (2.03% - 3.20%)	0.17% (0.05% - 0.39%)	0.07% (0.01% - 0.24%)	0.00% (0.00% - 0.12%)	0.00% (0.00% - 0.12%)
Durango	9,364	96.07% (95.66% - 96.45%)	1.17% (0.97% - 1.41%)	0.10% (0.04% - 0.18%)	0.14% (0.07% - 0.24%)	2.27% (1.98% - 2.60%)	0.23% (0.15% - 0.36%)	0.01% (0.00% - 0.06%)	0.00% (0.00% - 0.04%)	0.00% (0.00% - 0.04%)
Estado de México	118,348	96.13% (96.02% - 96.24%)	0.58% (0.54% - 0.63%)	0.07% (0.06% - 0.09%)	0.08% (0.07% - 0.10%)	2.89% (2.80% - 2.99%)	0.22% (0.19% - 0.24%)	0.01% (0.01% - 0.02%)	0.00% (0.00% - 0.00%)	0.01% (0.00% - 0.01%)
Guanajuato	27,730	96.14% (95.91% - 96.37%)	1.09% (0.97% - 1.22%)	0.05% (0.03% - 0.08%)	0.10% (0.06% - 0.14%)	2.38% (2.21% - 2.57%)	0.20% (0.15% - 0.26%)	0.02% (0.01% - 0.04%)	0.01% (0.00% - 0.03%)	0.00% (0.00% - 0.02%)
Guerrero	4,433	95.76% (95.12% - 96.33%)	1.22% (0.92% - 1.59%)	0.07% (0.01% - 0.20%)	0.20% (0.09% - 0.39%)	2.50% (2.06% - 3.01%)	0.20% (0.09% - 0.39%)	0.02% (0.00% - 0.13%)	0.00% (0.00% - 0.08%)	0.02% (0.00% - 0.13%)
Hidalgo	4,364	96.31% (95.71% - 96.85%)	0.57% (0.37% - 0.84%)	0.07% (0.01% - 0.20%)	0.11% (0.04% - 0.27%)	2.82% (2.35% - 3.35%)	0.11% (0.04% - 0.27%)	0.00% (0.00% - 0.08%)	0.00% (0.00% - 0.08%)	0.00% (0.00% - 0.08%)
Jalisco	33,441	95.51% (95.28% - 95.73%)	1.45% (1.32% - 1.58%)	0.08% (0.06% - 0.12%)	0.16% (0.12% - 0.21%)	2.44% (2.27% - 2.61%)	0.31% (0.25% - 0.38%)	0.02% (0.01% - 0.04%)	0.02% (0.01% - 0.05%)	0.01% (0.00% - 0.03%)
Michoacan	12,762	95.58% (95.21% - 95.93%)	1.26% (1.08% - 1.47%)	0.06% (0.03% - 0.12%)	0.12% (0.07% - 0.19%)	2.66% (2.39% - 2.96%)	0.24% (0.17% - 0.34%)	0.02% (0.00% - 0.07%)	0.03% (0.01% - 0.08%)	0.02% (0.00% - 0.06%)
Morelos	4,022	95.92% (95.26% - 96.51%)	0.47% (0.28% - 0.74%)	0.05% (0.01% - 0.18%)	0.25% (0.12% - 0.46%)	3.11% (2.59% - 3.69%)	0.17% (0.07% - 0.36%)	0.02% (0.00% - 0.14%)	0.00% (0.00% - 0.09%)	0.00% (0.00% - 0.09%)
Nayarit	5,059	95.43% (94.82% - 95.99%)	1.36% (1.06% - 1.72%)	0.08% (0.02% - 0.20%)	0.30% (0.17% - 0.49%)	2.41% (2.01% - 2.87%)	0.34% (0.20% - 0.54%)	0.06% (0.01% - 0.17%)	0.00% (0.00% - 0.07%)	0.02% (0.00% - 0.11%)
Nuevo León	31,376	96.91% (96.71% - 97.10%)	0.89% (0.79% - 1.00%)	0.05% (0.03% - 0.08%)	0.08% (0.05% - 0.12%)	1.86% (1.72% - 2.02%)	0.18% (0.13% - 0.23%)	0.01% (0.00% - 0.03%)	0.01% (0.00% - 0.02%)	0.00% (0.00% - 0.02%)
Oaxaca	3,845	94.67% (93.91% - 95.36%)	1.77% (1.38% - 2.24%)	0.10% (0.03% - 0.27%)	0.13% (0.04% - 0.30%)	3.09% (2.57% - 3.69%)	0.18% (0.07% - 0.37%)	0.03% (0.00% - 0.14%)	0.03% (0.00% - 0.14%)	0.00% (0.00% - 0.10%)
Puebla	33,474	95.25% (95.02% - 95.48%)	1.22% (1.11% - 1.35%)	0.08% (0.05% - 0.11%)	0.11% (0.08% - 0.16%)	3.02% (2.84% - 3.21%)	0.27% (0.22% - 0.33%)	0.04% (0.02% - 0.07%)	0.01% (0.00% - 0.03%)	0.00% (0.00% - 0.01%)
Querétaro	16,507	96.26% (95.96% - 96.55%)	1.05% (0.90% - 1.22%)	0.06% (0.03% - 0.11%)	0.06% (0.03% - 0.11%)	2.38% (2.15% - 2.62%)	0.16% (0.10% - 0.23%)	0.01% (0.00% - 0.04%)	0.01% (0.00% - 0.04%)	0.01% (0.00% - 0.03%)
Quintana Roo	8,185	95.53% (95.06% - 95.97%)	0.57% (0.42% - 0.76%)	0.11% (0.05% - 0.21%)	0.18% (0.10% - 0.30%)	3.19% (2.82% - 3.59%)	0.39% (0.27% - 0.55%)	0.02% (0.00% - 0.09%)	0.00% (0.00% - 0.05%)	0.00% (0.00% - 0.05%)
San Luis Potosí	7,723	97.23% (96.84% - 97.58%)	0.70% (0.53% - 0.91%)	0.04% (0.01% - 0.11%)	0.13% (0.06% - 0.24%)	1.71% (1.43% - 2.02%)	0.19% (0.11% - 0.32%)	0.00% (0.00% - 0.05%)	0.00% (0.00% - 0.05%)	0.00% (0.00% - 0.05%)
Sinaloa	28,451	96.07% (95.84% - 96.30%)	1.21% (1.09% - 1.34%)	0.05% (0.03% - 0.09%)	0.19% (0.15% - 0.25%)	2.12% (1.96% - 2.29%)	0.30% (0.24% - 0.37%)	0.02% (0.01% - 0.05%)	0.02% (0.01% - 0.05%)	0.01% (0.00% - 0.03%)
Sonora	19,103	96.04% (95.75% - 96.31%)	1.22% (1.07% - 1.39%)	0.04% (0.02% - 0.08%)	0.13% (0.08% - 0.19%)	2.21% (2.01% - 2.43%)	0.32% (0.25% - 0.42%)	0.01% (0.00% - 0.04%)	0.01% (0.00% - 0.04%)	0.01% (0.00% - 0.04%)
Tabasco	7,724	95.90% (95.43% - 96.33%)	0.54% (0.39% - 0.73%)	0.04% (0.01% - 0.11%)	0.05% (0.01% - 0.13%)	3.21% (2.83% - 3.63%)	0.23% (0.14% - 0.37%)	0.03% (0.00% - 0.09%)	0.00% (0.00% - 0.05%)	0.00% (0.00% - 0.05%)
Tamaulipas	14,112	97.00% (96.70% - 97.27%)	0.70% (0.57% - 0.85%)	0.06% (0.02% - 0.11%)	0.14% (0.09% - 0.22%)	1.76% (1.55% - 1.99%)	0.31% (0.23% - 0.42%)	0.01% (0.00% - 0.04%)	0.00% (0.00% - 0.03%)	0.03% (0.01% - 0.07%)
Tlaxcala	2,484	95.13% (94.21% - 95.94%)	0.68% (0.40% - 1.09%)	0.08% (0.01% - 0.29%)	0.00% (0.00% - 0.15%)	3.70% (3.00% - 4.52%)	0.40% (0.19% - 0.74%)	0.00% (0.00% - 0.15%)	0.00% (0.00% - 0.15%)	0.00% (0.00% - 0.15%)
Veracruz	24,430	95.79% (95.53% - 96.04%)	1.04% (0.91% - 1.17%)	0.06% (0.03% - 0.10%)	0.10% (0.07% - 0.15%)	2.63% (2.43% - 2.84%)	0.33% (0.26% - 0.41%)	0.02% (0.00% - 0.04%)	0.02% (0.01% - 0.05%)	0.01% (0.00% - 0.04%)
Yucatán	7,770	95.96% (95.50% - 96.39%)	0.51% (0.37% - 0.70%)	0.10% (0.04% - 0.20%)	0.08% (0.03% - 0.17%)	2.87% (2.51% - 3.27%)	0.46% (0.32% - 0.64%)	0.01% (0.00% - 0.07%)	0.00% (0.00% - 0.05%)	0.00% (0.00% - 0.05%)
Zacatecas	2,988	96.89% (96.20% - 97.48%)	1.17% (0.82% - 1.63%)	0.07% (0.01% - 0.24%)	0.23% (0.09% - 0.48%)	1.47% (1.07% - 1.97%)	0.13% (0.04% - 0.34%)	0.00% (0.00% - 0.12%)	0.03% (0.00% - 0.19%)	0.00% (0.00% - 0.12%)
**Total**	596,944	96.06% (96.01% - 96.11%)	0.89% (0.87% - 0.91%)	0.07% (0.06% - 0.07%)	0.11% (0.10% - 0.12%)	2.58% (2.54% - 2.62%)	0.26% (0.25% - 0.27%)	0.02% (0.01% - 0.02%)	0.01% (0.01% - 0.01%)	0.01% (0.00% - 0.01%)

Supplementary table 3. Cytological abnormalities prevalence in Mexico and its states. Abreviatures: CI = Confidence interval. NILM =Negative for intraepithelial lesions or malignancy, ASCUS = atypical squamous cells with undetermined significance, AGC= atypical glandular cells, ASCH= atypical squamous cells cannot exclude high-grade squamous intraepithelial lesions, LSIL= low-grade squamous intraepithelial lesions, HSIL= high-grade squamous intraepithelial lesions, SCC= squamous cancer cells, AIS= adenocarcinoma *in situ*, ADC= adenocarcinoma.

Age (years)	n	Prevalence of cytological abnormalities % (95% CI)								
		NILM	ASCUS	AGC	ASCH	LSIL	HSIL	SCC	AIS	ADC
<25	63,147	92.51% (92.30% - 92.71%)	1.65% (1.55% - 1.75%)	0.03% (0.01% - 0.04%)	0.05% (0.03% - 0.07%)	5.65% (5.47% - 5.83%)	0.12% (0.09% - 0.15%)	0.00% (0.00% - 0.01%)	0.00% (0.00% - 0.01%)	0.00% (0.00% - 0.01%)
25-34	139,288	94.98% (94.86% - 95.09%)	1.07% (1.02% - 1.13%)	0.07% (0.06% - 0.09%)	0.11% (0.10% - 0.13%)	3.40% (3.30% - 3.49%)	0.36% (0.32% - 0.38%)	0.01% (0.00% - 0.01%)	0.01% (0.00% - 0.01%)	0.00% (0.00% - 0.01%)
35-44	127,303	96.37% (96.27% - 96.47%)	0.78% (0.73% - 0.83%)	0.10% (0.08% - 0.12%)	0.11% (0.09% - 0.13%)	2.23% (2.15% - 2.31%)	0.37% (0.34% - 0.40%)	0.02% (0.02% - 0.03%)	0.01% (0.01% - 0.02%)	0.01% (0.00% - 0.02%)
45-54	145,101	96.94% (96.85% - 97.03%)	0.75% (0.70% - 0.79%)	0.06% (0.05% - 0.08%)	0.12% (0.10% - 0.14%)	1.92% (1.85% - 1.99%)	0.19% (0.17% - 0.22%)	0.01% (0.01% - 0.02%)	0.01% (0.00% - 0.01%)	0.01% (0.00% - 0.01%)
55-64	92,685	97.66% (97.56% - 97.76%)	0.60% (0.55% - 0.65%)	0.06% (0.04% - 0.07%)	0.12% (0.10% - 0.15%)	1.33% (1.26% - 1.41%)	0.19% (0.16% - 0.22%)	0.03% (0.02% - 0.04%)	0.00% (0.00% - 0.01%)	0.01% (0.00% - 0.02%)
65-74	26,253	98.13% (97.96% - 98.29%)	0.45% (0.38% - 0.54%)	0.03% (0.02% - 0.07%)	0.16% (0.12% - 0.22%)	0.94% (0.83% - 1.07%)	0.19% (0.14% - 0.25%)	0.05% (0.03% - 0.09%)	0.02% (0.01% - 0.04%)	0.01% (0.00% - 0.03%)
≥75	3,167	97.92% (97.36% - 98.38%)	0.73% (0.46% - 1.09%)	0.06% (0.01% - 0.23%)	0.03% (0.00% - 0.18%)	0.63% (0.39% - 0.97%)	0.47% (0.27% - 0.78%)	0.09% (0.02% - 0.28%)	0.00% (0.00% - 0.12%)	0.06% (0.01% - 0.23%)
TOTAL	596,944									

Supplementary table 4. Cytological abnormalities prevalence in Mexico by age. Abreviatures: CI = Confidence interval. NILM =Negative for intraepithelial lesions or malignancy, ASCUS = atypical squamous cells with undetermined significance, AGC= atypical glandular cells, ASCH= atypical squamous cells cannot exclude high-grade squamous intraepithelial lesions, LSIL= low-grade squamous intraepithelial lesions, HSIL= high-grade squamous intraepithelial lesions, SCC= squamous cancer cells, AIS= adenocarcinoma *in situ*, ADC= adenocarcinoma.

	NILM		ASCUS		AGC		ASCH		LSIL		HSIL		SCC		AIS		ADC	
	n	% (95% CI)	n	% (95% CI)	n	% (95% CI)	n	% (95% CI)	n	% (95% CI)	n	% (95% CI)	n	% (95% CI)	n	% (95% CI)	n	% (95% CI)
HPV (+)	112135	19.55% (19.45% - 19.66%)	3432	64.67% (63.37% - 65.96%)	244	61.93% (56.93% - 66.74%)	481	73.55% (69.99% - 76.89%)	12269	79.61% (78.97% - 80.25%)	1448	93.12% (91.75% - 94.33%)	88	89.80% (82.03% - 95.00%)	44	88.00% (75.69% - 95.47%)	22	56.41% (39.62% - 72.19%)
HPV (-)	461301	80.45% (80.34% - 80.55%)	1875	35.33% (34.04% - 36.63%)	150	38.07% (33.26% - 43.07%)	173	26.45% (23.11% - 30.01%)	3142	20.39% (19.75% - 21.03%)	107	6.88% (5.67% - 8.25%)	10	10.20% (5.00% - 17.97%)	6	12.00% (4.53% - 24.31%)	17	43.59% (27.81% - 60.38%)
HPV 16	16984	2.96% (2.92% - 3.01%)	551	10.38% (9.57% - 11.23%)	72	18.27% (14.58% - 22.45%)	149	22.78% (19.62% - 26.19%)	1747	11.34% (10.84% - 11.85%)	578	37.17% (34.76% - 39.63%)	47	47.96% (37.76% - 58.29%)	24	48.00% (33.66% - 62.58%)	10	25.64% (44.62% - 76.64%)
HPV 18	8874	1.55% (1.52% - 1.58%)	286	5.39% (4.80% - 6.03%)	44	11.17% (8.23% - 14.70%)	46	7.03% (5.20% - 9.27%)	910	5.90% (5.54% - 6.29%)	150	9.65% (8.22% - 11.22%)	14	14.29% (8.04% - 22.81%)	9	18.00% (8.58% - 31.44%)	6	15.38% (5.86% - 30.53%)
HPV POOL	96650	16.85% (16.76% - 16.95%)	3036	57.21% (55.86% - 58.54%)	178	45.18% (40.19% - 50.24%)	373	57.03% (53.14% - 60.86%)	11354	73.67% (72.97% - 74.37%)	1031	66.30% (63.89% - 68.65%)	40	40.82% (30.99% - 51.21%)	23	46.00% (31.81% - 60.68%)	10	25.64% (13.04% - 42.13%)

Supplementary table 5. Cytological abnormalities correlation to HPV infection in Mexico. Abreviatures: CI = Confidence interval. NILM =Negative for intraepithelial lesions or malignancy, ASCUS = atypical squamous cells with undetermined significance, AGC= atypical glandular cells, ASCH= atypical squamous cells cannot exclude high-grade squamous intraepithelial lesions, LSIL= low-grade squamous intraepithelial lesions, HSIL= high-grade squamous intraepithelial lesions, SCC= squamous cancer cells, AIS= adenocarcinoma *in situ*, ADC= adenocarcinoma, HPV = Human papillomavirus.

Vaccination status	n	%	Prevalence of HPV infections % (95% CI)			
			Overall	HPV 16	HPV 18	HPV POOL
Non vaccinated	36,865	58.38%	37.98% (37.07% - 38.48%)	3.17% (2.99% - 3.35%)	1.24% (1.13% - 1.36%)	36.21% (35.71% - 36.70%)
1 dose	10,960	17.36%	37.36% (36.49% - 38.27%)	1.68% (1.44% - 1.92%)	0.78% (0.61% - 0.94%)	36.41% (35.51% - 37.32%)
2 doses	11,730	18.58%	36.09% (34.52% - 36.96%)	0.68% (0.53% - 0.83%)	0.41% (0.29% - 0.52%)	35.64% (34.77% - 36.50%)
3 doses	3,592	5.69%	35.77% (35.28% - 37.34%)	0.58% (0.34% - 0.83%)	0.25% (0.09% - 0.41%)	35.47% (33.90% - 37.03%)
Total	63,147	100.00%				

* Women with an age of 25 years or older were excluded from the analysis

Supplementary table 6. Overall HPV, HPV 16, HPV 18, and HPV POOL genotypes prevalence in vaccinated and non-vaccinated women. Abreviatures: CI = Confidence interval. HPV = Human papillomavirus.

Characteristic	n	HPV Positive (%)	Age adjusted OR (95% CI)	*p* Value
**Vaccination (HPV)**				
1 dose	10,960	37.36%	0.97 (0.93-1.02)	0.241
2 doses	11,730	36.09%	0.92 (0.88-0.96)	<0.001
3 doses	3,592	35.77%	0.91 (0.85-0.98)	<0.05
No	36,865	37.98%	1	
**Vaccination (HPV 16)**				
1 dose	10,960	1.68%	0.52 (0.44-0.61)	<0.0001
2 doses	11,730	0.68%	0.21 (0.17-0.26)	<0.0001
3 doses	3,592	0.58%	0.18 (0.11-0.26)	<0.0001
No	36,865	3.17%	1	
**Vaccination (HPV 18)**				
1 dose	10,960	0.78%	0.62 (0.49-0.78)	<0.0001
2 doses	11,730	0.41%	0.33 (0.24-0.44)	<0.0001
3 doses	3,592	0.25%	0.20 (0.10-0.36)	<0.0001
No	36,865	1.24%	1	
**Vaccination (HPV POOL)**				
1 dose	10,960	36.41%	1.01 (0.97-1.05)	0.689
2 doses	11,730	35.64%	0.98 (0.93-1.02)	0.262
3 doses	3,592	35.47%	0.97 (0.90-1.04)	0.380
No	36,865	36.21%	1	

Supplementary table 7. Vaccine protection análisis in Mexican women younger than 25. Odds ratio were obtained from stepwise multilevel logistic regression equations. Abreviatures: CI = Confidence interval. HPV = Human papillomavirus.
